# Comprehending Body Language and Mimics: An ERP and Neuroimaging Study on Italian Actors and Viewers

**DOI:** 10.1371/journal.pone.0091294

**Published:** 2014-03-07

**Authors:** Alice Mado Proverbio, Marta Calbi, Mirella Manfredi, Alberto Zani

**Affiliations:** 1 Department of Psychology, University of Milano-Bicocca, Milan, Italy; 2 Department of Neuroscience, University of Parma, Parma, Italy; 3 Department of Cognitive Science, University of California San Diego, La Jolla, San Diego, California, United States of America; 4 Institute of Molecular Bioimaging and Physiology (IBFM), National Research Council (CNR), Milan, Italy; University of Bologna, Italy

## Abstract

In this study, the neural mechanism subserving the ability to understand people’s emotional and mental states by observing their body language (facial expression, body posture and mimics) was investigated in healthy volunteers. ERPs were recorded in 30 Italian University students while they evaluated 280 pictures of highly ecological displays of emotional body language that were acted out by 8 male and female Italian actors. Pictures were briefly flashed and preceded by short verbal descriptions (e.g., “What a bore!”) that were incongruent half of the time (e.g., a picture of a very attentive and concentrated person shown after the previous example verbal description). ERP data and source reconstruction indicated that the first recognition of incongruent body language occurred 300 ms post-stimulus. swLORETA performed on the N400 identified the strongest generators of this effect in the right rectal gyrus (BA11) of the ventromedial orbitofrontal cortex, the bilateral uncus (limbic system) and the cingulate cortex, the cortical areas devoted to face and body processing (STS, FFA EBA) and the premotor cortex (BA6), which is involved in action understanding. These results indicate that face and body mimics undergo a prioritized processing that is mostly represented in the affective brain and is rapidly compared with verbal information. This process is likely able to regulate social interactions by providing on-line information about the sincerity and trustfulness of others.

## Introduction

Social interactions are based on the transmission of both verbal and non-verbal information, which are automatically processed in parallel. Evidence has been provided that suggests that we are more impressed by the implicit (non-verbal) than the explicit information we receive. Indeed, in contrast to people’s verbal statements, people’s intentions and beliefs can be inferred from how they move their bodies or modulate their facial mimicry [1]–[2].

Indeed, kinematic studies have identified what cues observers rely on for detection of social intentions (e.g., [3]). Notably, the faster we can judge other’s intentions, the more time we have to select a suitable response [4].

It is well understood that non-verbal behavior and “emotional body language” (EBL) have crucial roles in communication and guiding social interactions [5], however not much is known about the neural underpinnings of this complex ability, especially compared to the large numbers of neuroscientific investigations of explicit linguistic communication that have been carried out on explicit linguistic communication.

It is known that visual processing of the human body and its emotional displays (that are based on motion and mimicry) activates brain regions that are normally involved in the processing of face and body structural properties [6]–[10] such as the face fusiform area (FFA) [11], the extra-striate body area (EBA) [12], which is located at the posterior inferior temporal sulcus/middle temporal gyrus, and the fusiform body area (FBA) [13], which is found ventrally in the fusiform gyrus; all of these areas normally operate in concert with the amygdala and the superior temporal sulcus (STS).

Peelen and coworkers [14] measured the degrees of activation of the EBA and FBA in response to “emotional” and neutral body language. The authors presented short movie clips of people expressing 5 basic emotions (anger, disgust, fear, happiness, and sadness) or performing emotionally neutral gestures. The results showed that the functionally localized EBA and FBA were influenced by the emotional significance of body movements. Furthermore, using multi-voxel pattern analysis, these authors showed that the activities of these two regions were not only greater in response to emotional versus neutral bodies but also that such emotion-related increases correlated positively with the degree of body selectivity across voxels. Similarly, De Gelder and coworkers [15] contrasted brain activations during the perception of frightened or happy EBL. Affective images and images of neutral body movements were alternately displayed, and the faces of the actors were obscured. The results revealed increased BOLD signals in areas responsible for the processing of emotions, including the amygdala and the orbitofrontal cortex (OFC), and in motor areas, such as the premotor cortex.

Amoruso and coworkers [16] recently proposed an integrated functional neuroanatomic model of EBL and action meaning comprehension in which the EBA and FBA provide perceptual information about people and their interactions that is integrated into a larger fronto-insular–temporal network. More specifically, this network includes the following components: several frontal areas that update and associate ongoing contextual information in relation to episodic memory, the STG, the parahippocampal cortex (PHC), the hippocampus, and the amygdala, which indexes the value of learning target-context associations (affective information). Additionally, in this proposed model, the insular cortex coordinates internal and external milieus with an inner motivational state. An interesting functional magnetic resonance imaging study has provided direct evidence that the EBA is not only highly responsive when subjects observe isolated faces presented in emotional scenes but also highly responsive to threatening scenes in which no body is present [17]; these findings suggest that the role of the EBA in EBL comprehension extends beyond the processing of body structures.

Despite the incredible complexity of the non-facial mimicry and gestures that humans (especially Mediterranean people such as Italians) use to communicate their emotional and mental states, neuroimaging investigations (described above) have thus far dealt solely with basic affective emotions (e.g., anger, happiness, fearfulness, and disgust) and have primarily been based on facial expressions or a limited set of stereotyped symbolic gestures (e.g., indicating “victory” with 2 fingers [18]) or stick figure characters [19] that are not ecologically relevant.

To address this issue, we created a large set of highly ecological and complex body language patterns by taking pictures of real Italian actors impersonating emotional states in front of a camera according to the Stanislavski method. This method is based not only on character’s psychological analysis, but also on a personal research between character’s interior world and the actor’s one. It concerns the expression of interior emotions through their interpretation to enable actors to draw believable emotions to their performances [20].

All actions and gestures used in this study reflected the actors’ emotional (or physiological) states, rather than a neutral semantic meaning (e.g.: “drinking”, “driving”, “smoking”, etc.). Therefore they represented people emotional body language (EBL). To measure the neural processing associated with EBL comprehension, the neural processing of body language patterns preceded by congruent descriptions of the feeling displayed (e.g., “Come here, let me hug you!” followed by a picture of a person with a big smile and open arms) or incongruent description (e.g., “I hate you!”) were compared. We hypothesized that presenting a verbal description of an emotional or physiological state would activate the conceptual representation of corresponding body language (because of resonating empathic systems), and that the presentation of a picture representing a person actually experiencing the same or totally different feeling would stimulate a congruent (“same”) vs. incongruent (“different”) neural response. Electric neuroimaging literature have identified such a response as a negative deflection peaking at about 400 ms (but generally more anterior than linguistic N400) indexing the automatic detection of an incongruence between incoming visual information about an action being performed, and previous knowledge (about the action’s goal, intention, appropriateness, procedure, context of use, etc.): [16], [21]–[25].

In this study, ERPs were recorded in response to nearly 300 pictures of male and female actors displaying clearly recognizable EBL (as previously validated by a group of judges) in the 2 conditions. Pictures were carefully matched across categories for perceptual and sensory characteristics (such as size, luminance, color, body characteristics, body position, body orientation, clothes, body region involved in the mimicry, etc.). We therefore assumed that any differences in the ERP response amplitudes (especially the N400) at any site or latency could be interpreted as bioelectric indexes of the neural activity linked to the recognition or the detection discrepancies between prior verbal descriptions of an affect and the recognition of an affect expressed by the perceived body language. Source reconstruction was applied to the surface potentials to identify the neural generators responsive to incongruence; thus, spatial resolution was added to the optimal millisecond resolution provided by this electrophysiological technique.

## Methods

### Participants

Thirty healthy right-handed Italian University students (15 males and 15 females) were recruited for this experiment. Their ages ranged from 18 to 29 years (mean = 23 years; men = 24.27 SD = 2.37; women = 21.73 SD = 2.43). All had normal or corrected to normal vision and reported no history of neurological illness or drug abuse. Their handedness was assessed by the Italian version of the Edinburgh Handedness Inventory, which is a laterality preference questionnaire that reported strong right-handedness and right ocular dominance in all participants. Data from all participants were included in all analyses. Experiments were conducted with the understanding and written consent of each participant according to the Declaration of Helsinki (BMJ 1991; 302∶1194) with approval from the Ethical Committee of the Italian National Research Council (CNR) and in compliance with APA ethical standards for the treatment of human volunteers (1992, American Psychological Association).

### Stimuli and Materials

#### Stimulus validation

Stimulus materials were generated by taking ecological pictures of emotional body postures. Eight semi-professional actors (4 males and 4 females) were asked to display particular moods or emotional states using their entire body. The individual in this manuscript has given written informed consent (as outlined in PLOS consent form) to publish these case details. Photographs were taken in a classroom while the actors stood in front of the camera in a black hall in light-controlled conditions. A set of standardized instructions was given to each actor indicating that they should spontaneously express 40 typical emotional/mental states (listed in Table 1). The expressions of these emotional/mental states did not include symbolic or language-based gestures. For each of the 40 body-language categories, 8 pictures were taken, which resulted in a total of 320 pictures. Half of these pictures were assigned to the congruent condition, and the other half were assigned to the incongruent condition. In the congruent condition, the pictures were congruent with verbal descriptions that summarized the body language and immediately preceded the display of the pictures; in the incongruent condition, the pictures were incongruent with the verbal descriptions that immediately preceded them. Example verbal descriptions are provided in Table 1. The complexity of verbal description and emotional connotation of body-language categories was balanced across the congruent and incongruent classes, as shown in Table 2.

**Table 1:** List of the 40 emotional or mental stated portrayed by the actors and examples of the verbal labels used.

CONGRUENT	%	INCONGRUENT	%
**Anger**: “I am gonna smash your face!”	91	**Abandonment**: “Ah, what a relax…”	100
**Anxiety**: “Christ, what I’ve done!”	93	**Admiration**: “I admire you”	99
**Boredom**: “What a bore!”	96	**Approval**: “It’s right, well done!”	94
**Disgust**: “How disgusting!”	96	**Calmness**: “Ah, what a calm”	96
**Disinterest**: “I’m not interested!”	84	**Cold**: “It’s too cold”	99
**Desperation**: “Oh my God, noo!”	96	**Doubt**: “I have a doubt.”	99
**Endanger/Threat**: “Come here if you dare!”	93	**Ecstasy/Delight**: “I’m in seventh heaven”	100
**Fear**: “Help!”	86	**Guilt**: “I feel guilty”	99
**Happiness**: “I’m so happy!”	99	**Hate**: “I hate you.”	99
**Invoke/Beg**: “Oh Zeus!”	99	**Impatience**: “I’m so impatient.”	91
**Love:** “You’re so lovely/How lovely you’re”	94	**Regret**: “I feel remorse”	94
**Sadness**: “ I’m so sad…”	96	**Repugnance**: “You are disgusting!”	95
**Seduction**: “Do you like me?/Come here, babe.!”	99	**Revenge**: “You’ll pay back”	82
**Shame/Embarrassment:** “I feel so ashamed”	93	**Shyness**: “I’m shy”	100
**Surprise:** “ Wow, I can’t believe it!”	94	**Suspicion**: “Go away!”	93
**Tiredness**: “I’m so tired…”	97	**To be proud of**: “I’m so beautiful…”	98
**To pray**: “I implore you!”	100	**To explain**: “It’s funny, let me explain you.”	98
**To reprimand**: “Don’t do that! Go in disgrace!”	97	**To kid**: “You make me laugh!”	99
**To think**: “Let me think about it.”	100	**To love s.o**.: “I love you”	97
**Victory**: “Yes, scooore!!”	100	**To play up to (s.o.)**: “You are so sweet”	99

Notice that, in the incongruent condition (right column), the verbal descriptions (provided below)were not compatible with the emotional states displayed. Emotional categories are accompanied by the average percentage of correct recognitions obtained in the validation task (N° of judges = 12). Accuracy was 95.3% for pictures assigned to the congruent and 96% for pictures assigned to the incongruent condition.

**Table 2:** Average number of words and verbs (along with standard deviations) contained in verbal descriptions preceding pictorial stimuli.

	N° of words	SD	N° of verbs	SD	POSITIVE	NEGATIVE	NEUTRAL
**CONGRUENT**	3.28	1.26	0.96	0.69	5 (25%)	12 (60%)	3 (15%)
**INCONGRUENT**	2.90	0.91	0.90	0.45	6 (30%)	10 (50%)	4 (20%)

Verbal complexity was matched across classes, as well as the number of positive, negative or neutral body language categories assigned to congruent vs. incongruent trials.

To test the validity of the pictures (i.e., to ensure that they were easily comprehensible in terms of their intended meanings), they were presented to a group of 12 judges (8 women, 4 men) with a mean age of 29.9 years. These judges were asked to judge the coherence between the EBL of the pictures and the verbal labels associated with them. Specifically, the judges were asked, “How likely is it that the person pictures would actually think or say something like that?” The judges responded by pressing a button to signal “Yes, it’s likely” (congruent) or another button to signal “No, it’s not likely” (incongruent).

All pictures were randomly ordered one per page in a PowerPoint file with their associated verbal descriptions and presented to the 12 judges. The experimenter showed the judges the pictures one by one for a few seconds each and asked them to rapidly evaluate the congruency as described above. Only pictures that were evaluated consistently by at least 75% of the judges were included in the experimental set; the other pictures were rejected or the corresponding verbal descriptions were changed.

#### Final stimuli for ERP experiment

**Figure 1:**
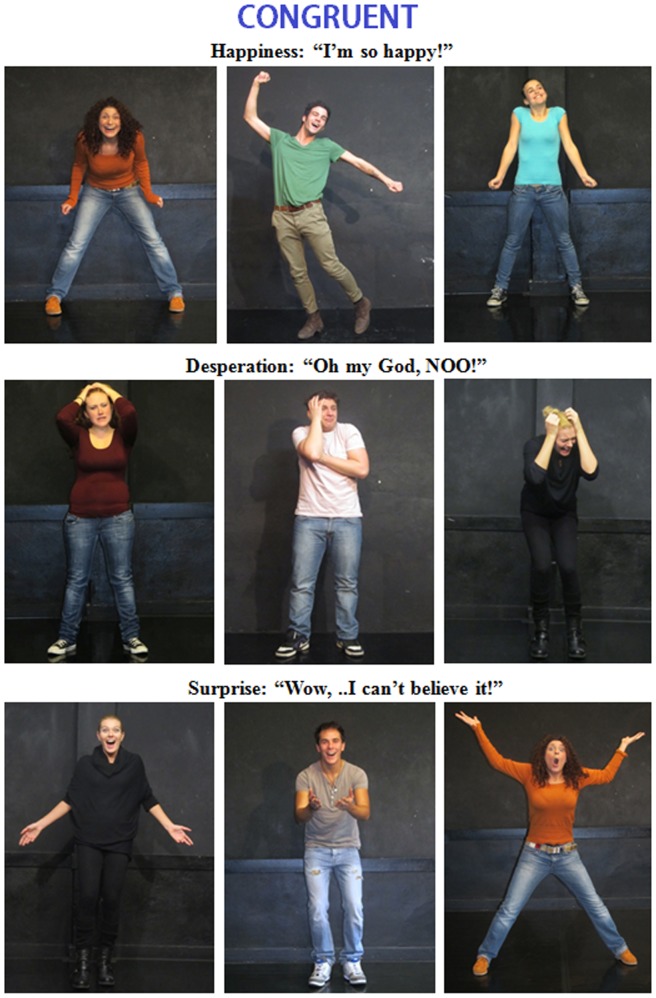
Examples of the body expressions portrayed by the male and female actors and CONGRUENT emotional or mental states.

**Figure 2:**
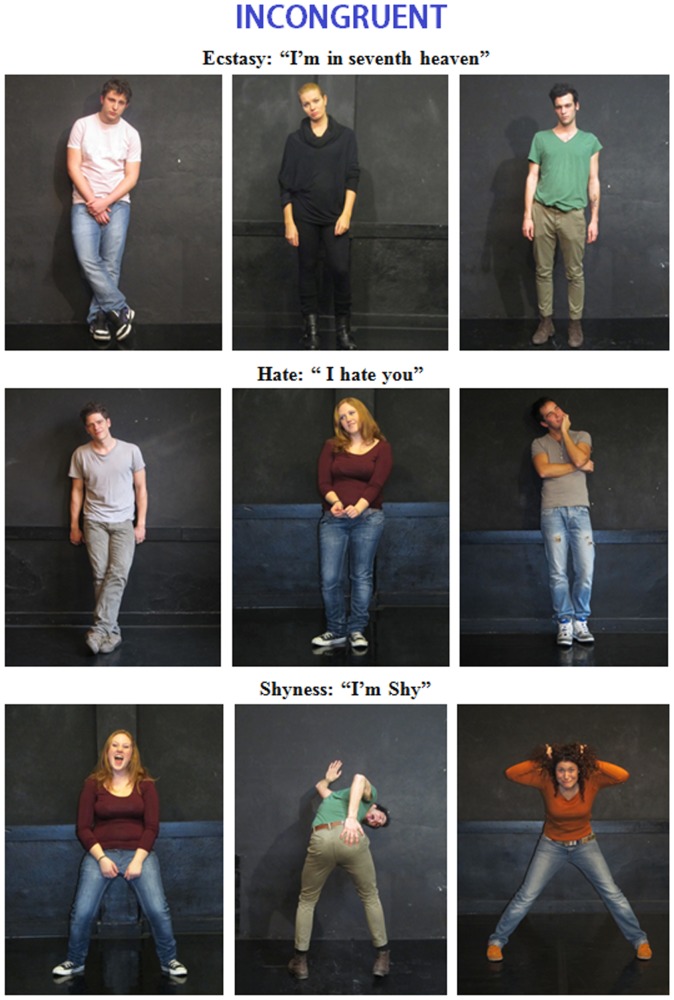
Examples of the body expressions portrayed by the male and female actors and INCONGRUENT emotional or mental states.

At the end of this process, we selected 280 pictures (half were congruent, and half were incongruent). Figures 1 and 2 show example stimuli for the various emotional states. In visual angle, the stimuli were 6° in length and 8° in height. The stimuli were equiluminant: an ANOVA revealed no difference in picture luminance across the categories (congruent = 9.33 cd/cm2; incongruent = 8.93 cd/cm2). The verbal descriptions were presented in Arial Narrow font and were written in white on a black background. The lengths of these descriptions ranged from 3 to 11 cm, which subtended visual angles of 1° 30′ to 5° 30′ on the horizontal axis. The heights of the descriptions ranged from 1 to 4 cm, which subtended visual angles of 30′ to 2° on the vertical axis. Each verbal description was presented in short lines (1 to 3 words per line) for 700 ms at the center of the PC screen with inter-stimulus intervals (ISIs) that ranged from 100 to 200 ms and were followed by the corresponding picture, which was presented for 1200 ms with an ISI of 1500 ms. The outer background was black.

### Procedure

The task consisted of responding as accurately and quickly as possible to the pictures judged to be congruent by pressing a response key with the index finger (of the left or right hand) and to the pictures judged to be incongruent by pressing a response key with the middle finger (of the left or right hand). The hand used was alternated during the recording session (to avoid possible biases due to the prolonged activation of the contralateral hemisphere). Hand orders and the task conditions were counterbalanced across subjects. At the beginning of each session, the subjects were told which hand would be used to indicate their responses.

The participants were seated comfortably in a darkened, acoustically and electrically shielded test area. They faced a high-resolution VGA computer screen located 114 cm from their eyes and were instructed to gaze at the center of the screen, where a small blue circle served as a fixation point, and to avoid any eye or body movements during the recording session. Stimuli were presented in a random order at the center of the screen in 8 blocks of 33–38 trials that lasted about 3 minutes each. Each block was preceded by a warning signal (a red cross) that was presented for 700 ms. The experimental session was preceded by a training session that included two runs, one for each hand. The sequence presentation order varied across subjects. The experiment lasted about 1 hour and a half (pauses included).

### EEG Recording and Analysis

The EEG was continuously recorded from 128 scalp sites at a sampling rate of 512 Hz. Horizontal and vertical eye movements were also recorded. Linked mastoids served as the reference lead. The EEG and electro-oculogram (EOG) were amplified with a half-amplitude band pass of 0.016–70 Hz. Electrode impedance was maintained below 5 kΩ. The EEG was recorded and analyzed using *EEProbe* recording software (*ANT Software*, Enschede, The Netherlands). Stimuli presentation and triggering was performed using *Eevoke* Software for audiovisual presentation (*ANT Software*, Enschede, The Netherlands).

EEG epochs were synchronized with the onset of stimuli presentation. A computerized artifact rejection criterion was applied before averaging to discard epochs in which eye movements, blinks, excessive muscle potentials or amplifier blocking occurred. The artifact rejection criterion was a peak-to-peak amplitude exceeding 50 µv, and the rejection rate was ∼5%. ERPs were averaged off-line from −100 ms before to 1200 ms after stimulus onset. ERP components were identified and measured with reference to the average baseline voltage over the interval of −100 to 0 ms at the sites and latencies at which they reached their maximum amplitudes. The choice of electrode sites and time windows for measuring and quantifying ERP components of interest was based both on previous literature and on the determination of when and where (on scalp surface) they reached their maximum values.

The mean amplitude (at peak) and latency of the posterior P300 response was measured at centroparietal (CP1, CP2) and occipitotemporal (P9, P10, PPO1, POO2) sites between 280 and 440 ms. The anterior N400 mean area amplitude was quantified at dorsolateral (F1, F2) and inferior (F5, F6) frontal sites in the 380–460 ms time window. The mean area amplitude of the centro-parietal N400 response was measured at the P1, P2, CPP1h, and CPP2h sites between 400 and 600 ms. The amplitude of the *late positivity* (LP) was measured over the occipitotemporal P9, P10, PPO1, PPO2 sites in the 650–850 ms time window.

ERP data were subjected to multifactorial repeated-measures ANOVAs with three within group factors: Condition (Congruent, Incongruent), Electrode (dependent upon the ERP component of interest) and Hemisphere (left, right). Multiple comparisons of the means were performed with Tukey’s post-hoc tests.

Topographical voltage maps of the ERPs were made by plotting color-coded isopotentials obtained by interpolating voltage values between scalp electrodes at specific latencies. Low-resolution electromagnetic tomography (LORETA; Pasqual-Marqui and coworkers [26]) was performed on the ERP waveforms from the anterior N400 (380–460 ms) using *ASA4 Software* (ANT Software, Enschede, The Netherlands).

Source reconstruction was performed on surface potentials recorded in the latency range of anterior N400, because it represented the first ERP modulation related to action content, and based on previous literature showing a modulation of the anterior N400 indexing the detection/discrimination of incongruent vs. congruent actions [18], [21], [22], [27], [28]. LORETA is a discrete linear solution to the inverse EEG problem, and it corresponds to the 3D distribution of neuronal electric activity that maximizes similarity (i.e., maximizes synchronization) in terms of orientation and strength between neighboring neuronal populations (represented by adjacent voxels). In this study, an improved version of standardized weighted low-resolution brain electromagnetic tomography (sLORETA) was used; this version incorporates a singular value decomposition-based lead field weighting (i.e., swLORETA; Palmero-Soler and coworkers [29]. The source space properties included a grid spacing (the distance between two calculation points) of 5 points and an estimated signal-to-noise ratio, which defines the regularization, of 3 (higher values indicating less regularization and therefore less blurred results). SwLORETA was performed on the group data and identified statistically significant electromagnetic dipoles (p<0.05); increases in the magnitudes of these dipoles correlated with more significant activation. The strength of a locus of activation is represented by the magnitude (magn.) of the electromagnetic signal (in nA m^−1^). The electromagnetic dipoles are shown as arrows and indicate the position, orientation and magnitude of dipole modeling solutions applied to the ERP waveform in the specific time window. The larger the magnitude, the more significantly a source was found to explain/contribute to the surface potential.

A realistic boundary element model (BEM) was derived from a T1-weighted 3D MRI data set by segmenting the brain tissue. This BEM model consisted of one homogenous compartment comprised of 3,446 vertices and 6,888 triangles. The head model was used for intracranial localization of surface potentials. Both segmentation and generation of the head model were performed using ASA software.

Reaction times (RTs) that exceeded the mean value ±2 standard deviations were discarded, which resulted in a rejection rate of 2%. Error rate percentages were converted to arcsin values. Both RTs and error percentages were subjected to separate multifactorial repeated-measures ANOVAs with 1 between-subject factor (gender: male or female) and 2 within-subject factors (condition: congruent or incongruent; and response hand: left or right).

## Results

### Behavioral Results

Analysis of the reaction times (RTs) revealed a main effect of response hand (F1, 28 = 9.1, p<0.0055) that was due to the responses of the right hand (828 ms, SE = 22) being faster than those of the non-dominant hand (851 ms, SE = 21). Neither gender nor stimulus congruence significantly affected RTs. The accuracy data indicated that fewer errors were committed in response to incongruent pictures (7.7%, SE = 1.5. Raw value = 2%) than in response to congruent pictures (20.9%, SE = 1.7. Raw value = 12%), and the corresponding main effect of congruence was significant (F1, 28 = 41.8, p<0.0055). No other factors significantly affected accuracy.

### Electrophysiological Data

**Figure 3:**
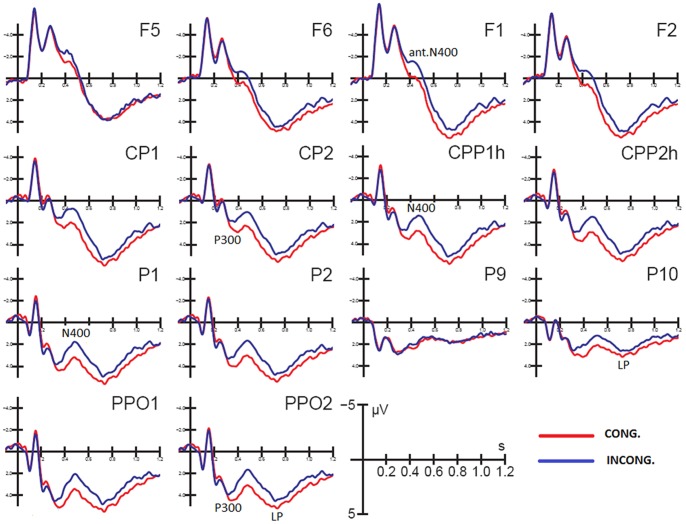
Grand-average ERP waveforms recorded at the left and right dorsolateral frontal (F5, F6) inferior frontal (F1, F2), centroparietal (CP1, CP2, CPP1h, CPP2h), parietal (P1, P2), occipitotemporal (P9, P10), and lateral occipital (PPO1h, PPO2h) electrode sites in response to Congruent and Incongruent images.

Figure 3 shows grand-average ERP waveforms recorded at various anterior and posterior sites as a function of the congruence of the actions and verbal description. A strong posterior modulation of the synchronized response that indicates the early recognition of expected gestures (as early as 280 ms and indexed by the P300 component) is visible. This modulation was followed by a centro/parietal N400 that was elicited by incongruent gestures (400–600 ms) and by a larger late positivity (LP) that was elicited by congruent gestures (650–850 ms). At the frontal sites, incongruent EBL was recognized as such as early as 380 ms (380–460 ms) as indexed by the large inferior frontal N400 response.

### Posterior Sites

#### P300 (280–440 ms)

The ANOVA performed on the peak amplitude values of the P300 revealed a significant effect of Condition (F(1,29) = 17.563; p<0.00024) that was driven by a stronger response to congruent (4.41 µv, SE = 0.43) than incongruent (3.82 µv, SE = 0.41) EBL. The significant effect of Electrode (F(2,58) = 17.627; p<0.000001) was driven by the presence of a larger P300 at lateral occipital (5.60 µv, SE = 0.59) than centro-parietal (3.22 µv, SE = 0.50) and occipitotemporal (3.51 µv, SE = 0.32) sites. The ANOVA also revealed a significant Condition × Electrode interaction (F(2,58) = 5.096; p<0.00915) that was driven by larger P300 responses to congruent images at posterior sites (5.96 µv, SE = 0.61; post-hoc comparisons: p<0.00052). The significant Condition × Hemisphere interaction (F(1,29) = 6.628; p<0.0155) was driven by much larger responses to congruent body expressions over the right (4.50 µv, SE = 0.42) than the left (4.32 µv, SE = 0.46) hemisphere (post-hoc tests: p<0.0123).

#### Latency

Latency analyses indicated that P300 occurred earlier for incongruent (340 ms, SE = 0.005) than congruent stimuli (359 ms, SE = 0.005) as indicated by the significant main effect of Condition (F(1,29) = 18.770; p<0.00017). This result was most likely related to differences in P300 amplitude since large and slow components (such as P300) typically reach their maximum amplitude later in time: the smaller, the earlier.

#### N400 (400–600 ms)

The ANOVA performed on the mean amplitudes of the posterior N400 revealed a significant effect of Condition (F(1,29) = 33,86; p<0.000004) that was driven by greater responses to incongruent (1.97 µv; SE = 0.33) than congruent EBL (3.71 µv; SE = 0.39). The main effect of electrode was also significant (F(2,58) = 10.34; p<0.00015) and was driven by larger N400s at occipitotemporal sites than parietal sites. However, the Condition × Electrode interaction (F(2,58) = 22.63; p<0.000001) was driven by the lack of effect of stimulus congruence over the visual (occipitotemporal) areas and the significantly increased N400 in response to incongruent pictures at central and centroparietal sites. The Hemisphere × Condition (F(1,29) = 4.48; p<0.05) and Hemisphere × Electrode × Condition interactions (F(2,58) = 10.964.48; p<0.00001) and the relevant post-hoc comparisons revealed a hemispheric asymmetry in the congruence effect; the effect was lacking at the left occipitotemporal sites but was significant over the right visual areas (P9: CONG 1.79 µv, SE = 0.29; INCONG 1. 56 µv; SE = 0.28. P10; CONG 2.43 µv, SE = 0.36; INCONG 1. 53 µv; SE = 0.34). The modulation of N400 in response to incongruent stimuli was significant at all sites (except P9) bilaterally, and this effect was larger at parietal sites (also visible in Fig. 3).

#### Late positivity (650–850 ms)

The LP was significantly affected by Condition (F(1,29) = 5.503; p<0.02604) and was of greater amplitude in response to congruent (3.63 µv, SE = 0.38) than incongruent (3.14 µv, SE = 0.41) EBL. The significant effect Electrode (F(1,29) = 86.731; p<0.000001) was driven by LPs at PPO1 and PPO2 (4.58 µv, SE = 0.47) than at P9 and P10 (2.19 µv, SE = 0.32). This result was confirmed by the significant Condition × Electrode interaction (F(1,29) = 8,410; p<0.00706), which was driven by greater responses at lateral occipital sites (PPO1, PPO2: CONG = 4.95 µv, SE = 0.48; INCONG = 4.21 µv, SE = 0.50) than at occipitotemporal (P9, P10: CONG = 2.31 µv, SE = 0.31; INCONG = 2.07 µv, SE = 0.35) sites (post-hoc comparisons: p<0.00017). The ANOVA also revealed a significance Condition × Hemisphere interaction (F(1,29) = 4.365; p<0.04556) that was driven by larger LP responses to congruent images over the right hemisphere (3.86 µv, SE = 0.46) compared to the left hemisphere (3.40 µv, SE = 0.35) (post-hoc comparison of the means: p<0.00325). The significant Electrode × Hemisphere interaction (F(1,29) = 8.290; p<0.00742) indicated larger LP amplitudes over lateral occipital (PPO1: = 4.69 µv, SE = 0.47; PPO2 = 4.47 µv, SE = 0.49) than occipitotemporal sites (P9 = 1.72 µv, SE = 0.25; P10 = 2.66 µv, SE = 0.47) and greater LP modulation over the right than over left hemisphere (post-hoc tests: p<0.01233).

The Condition × Electrode × Hemisphere (F(1,29) = 8.158; p<0.00785) interaction was driven by greater LP responses to Congruent (P9 = 1.74 µv, SE = 0.27; P10 = 2.89 µv, SE = 0.46; PPO1 = 5.06 µv SE = 0.48; PPO2 = 4.83 µv, SE = 0.50) than Incongruent (P9 = 1.71 µv, SE = 0.27; P10 = 2.42 µv, SE = 0.49; PPO1 = 4.31 µv, SE = 0.50; PPO2 = 4.12 µv, SE = 0.52) images particularly over the right lateral occipital sites (post-hoc comparisons: p<0.00028).

### Anterior Sites

#### N400 (380–460 ms)

**Figure 4:**
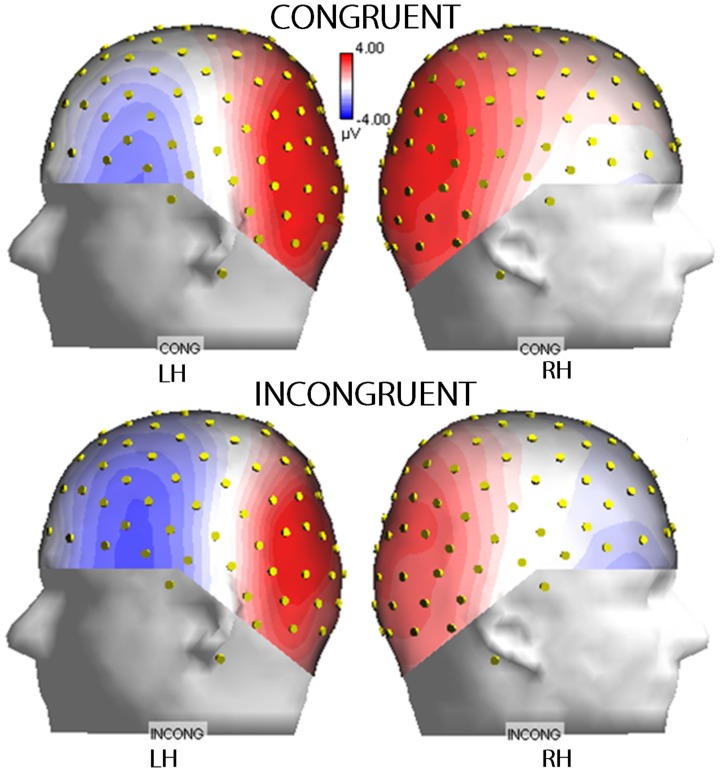
Isocolor topographical voltage maps obtained by plotting the N400 amplitudes recorded over the left (left column) and right (right column) hemispheres at a 400 ms latency.

The anterior N400 showed an effect of Condition (F(1,29) = 36.754; p<0.000001) that was driven by greater N400 responses to incongruent (−1.42 µv, SE = 0.49) than congruent images (−0.10 µv, SE = 0.47). The significant effect of Electrode (F(1,29) = 5.719; p<0.0235) was driven by greater N400 amplitudes at inferior frontal (−1.03 µv, SE = 0.43) sites than at frontal (−0.49 µv, SE = 0.52) sites (see also waveforms of Fig. 3). The ANOVA also revealed a significance effect of Hemisphere (F(1,29) = 31.755; p<0.00001) that was due to the larger N400 response over the left hemisphere (−1.38 µv, SE = 0.49) than over the right hemisphere (−0.14 µv, SE = 0.47). This result was confirmed by the significant Electrode × Hemisphere interaction (F(1,29) = 11,981; p<0.0017), which was due to the larger N400 response over the left (F1 = −0.87 µv, SE = 0.55; F5 = −1.89 µv, SE = 0.47) than over the right (F2 = −0.12 µv, SE = 0.53; F6 = −0.16 µv, SE = 0.44) hemispheric sites (post-hoc tests: p<0.00735). The significant Condition × Electrode interaction (F(1,29) = 13,499; p<0.00097) was due to the greater N400 modulatory effect of action incongruence at inferior frontal sites (INCONG = −1.58 µv, SE = 0.46; CONG = −0.47, SE = 0.43) compared to more dorsolateral frontal sites (INCONG = −1.26 µv, SE = 0.54; CONG = 0.26 µv, SE = 0.53). Both the topographic distribution and the left hemisphere asymmetry are clearly visible in the topographical maps in Fig. 4.

To locate the possible neural source of the N400 response, different swLORETA source reconstructions were performed on the brain voltages recorded in the Congruent and Incongruent conditions and the difference waves obtained by subtracting the ERPs elicited by the Congruent EBL from those elicited by the Incongruent EBL in the 380–460 ms time window. We assumed that while the processing of congruent EBL reflected the activation of the complex circuit for action, theory of mind, body and face analysis, body language processing and reading, etc., the processing of incongruent EBL specifically (and additionally) activated the regions more involved in the representation of supposed emotional state of others, besides regions representing a discrepancy in conceptual representation.

**Figure 5:**
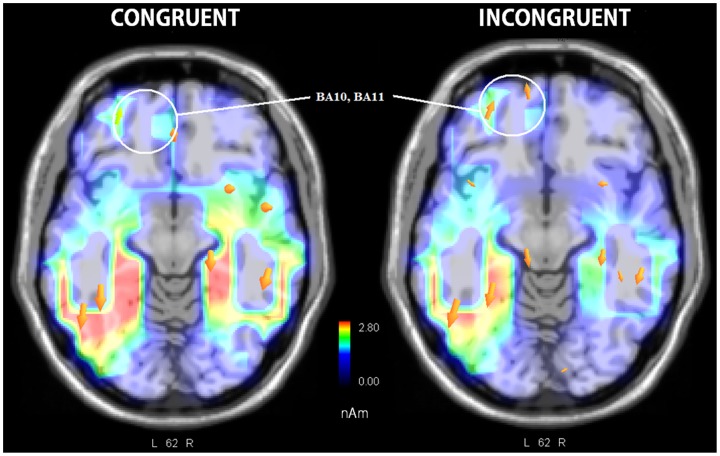
Axial views of the active N400 sources for congruent (left) and incongruent (right) brain waves according to swLORETA analysis during the 380–460 ms time window. The inverse solution was applied to the grand average signals (N = 30). The different colors represent differences in the magnitudes of the electromagnetic signals (in nAm). The electromagnetic dipoles are shown as arrows and indicate the position, orientation and magnitude of the dipole modeling solutions that were applied to the ERP waveforms in the specific time windows. The numbers refer to the displayed brain slice in the axial view: L = left, R = right.

**Figure 6:**
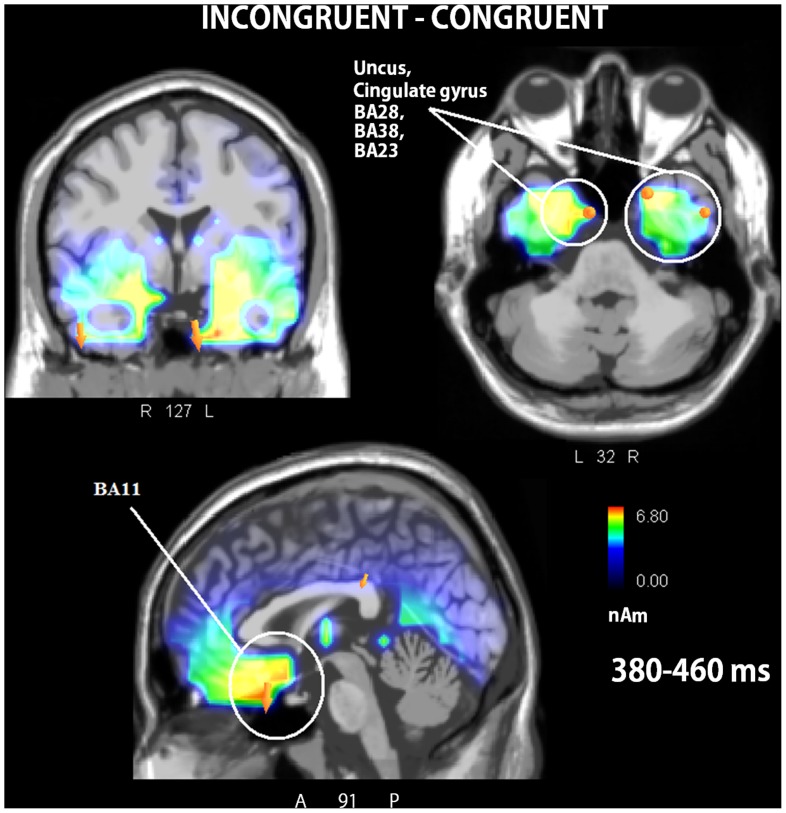
Coronal, axial and sagittal views of the N400 active sources relative to the difference wave (Incongruent – congruent) according to a swLORETA analysis that was applied to the 380–460 ms time window. A = anterior, P = posterior.

Table 3 shows the electromagnetic dipoles that significantly explained the surface voltages recorded in response to congruent (Top) and incongruent (Bottom) affective body language. A series of activations were common to the two conditions (clearly visible in Figure 5) and included the right (BA20) and left (BA37) fusiform gyri, the right parahippocampal gyrus (BA35), and the right supramarginal gyrus (BA40). The main differences between the congruent and incongruent conditions were the following: the activation of the right STG (BA38) elicited by congruent EBL (12.85 nAm) was stronger than that elicited by incongruent (11.73 nAm) EBL; the left postcentral gyrus of the parietal cortex was uniquely activated by congruent EBL; and the left premotor cortex was uniquely activated by incongruent EBL (BA6). To better appreciate the difference between the 2 conditions (since, naturally, the strongest signals came from face and body processing-devoted brain areas, commonly activated by congruent and incongruent EBL), a further swLORETA was applied to the grand-average difference-wave obtained by subtracting the ERPs elicited by congruent EBL from those elicited by incongruent EBL. Table 4 contains a list of significant sources, and the LORETA solution is visible in Figure 6. The processing of incongruent body language was associated with significant activities in the bilateral limbic (BA28, 38) and ventromedial orbitofrontal regions (BA11), and regions that are normally activated by human faces and bodies (BA 20, 21, 37).

**Table 3:** Talairach coordinates of the intracranial generators that explained the N400 surface voltages recorded in response to Congruent (Top) and Incongruent (Bottom) EBL images in the 380–460 ms window according to swLORETA inverse solution.

CONGRUENT							
Magn.	T - x	T - y	T - z	Hem	Lobe	Gyrus	BA
13.74	50.8	−33.7	−23.6	R	T	Fusiform Gyrus	20
13.52	−38.5	−44.8	−16.9	L	T	Fusiform Gyrus	37
13.43	−48.5	−58.9	14.5	L	O	Middle Temporal Gyrus	22
13.36	21.2	−24.5	−15.5	R	Limbic	Parahippocampal Gyrus	35
13.05	50.8	−0.6	−28.2	R	T	Middle Temporal Gyrus	21
12.85	31	9.1	−27.5	R	T	Superior Temporal Gyrus	38
12.46	1.5	38.2	−17.9	R	F	Medial Frontal Gyrus	11
12.23	−28.5	46.3	−2.3	L	F	Middle Frontal Gyrus	10
11.76	−38.5	−21	35.7	L	P	Postcentral Gyrus	3
11.52	40.9	−40.6	34	R	P	Supramarginal Gyrus	40
**INCONGRUENT**							
Magn.	T - x	T - y	T - z	Hem	Lobe	Gyrus	BA
13.53	−48.5	−59.9	23.4	L	T	Middle Temporal Gyrus	39
13.05	−28.5	−45.8	−9.5	L	Limbic	Parahippocampal Gyrus	37
12.56	50.8	−33.7	−23.6	R	T	Fusiform Gyrus	20
12.36	31	−24.5	−15.5	R	Limbic	Parahippocampal Gyrus	35
12.34	−28.5	46.3	−2.3	L	F	Middle Frontal Gyrus	10
12.2	−8.5	−30.4	34.9	L	Limbic	Cingulate Gyrus	31
11.95	−8.5	57.3	−9	L	F	Superior Frontal Gyrus	10
11.73	31	9.1	−27.5	R	T	Superior Temporal Gyrus	38
11.42	40.9	−40.6	34	R	P	Supramarginal Gyrus	40
11.24	−38.5	2.4	29.4	L	F	Precentral Gyrus	6

Power: 132.8 for the congruent condition and 128.9 for the incongruent condition.

**Table 4:** Talairach coordinates of the intracranial generators that explained the N400 surface difference-voltage (i.e., Incongruent minus Congruent EBL images) in the 380–460 ms window according to swLORETA. Power = 21.2.

INCONGRUENT- CONGRUENT							
Magn.	T - x	T - y	T - z	Hem	Lobe	Gyrus	BA
6.8	−8.5	−0.6	−28.2	L	Limbic	Uncus	28
6.75	21.2	9.1	−27.5	R	Limbic	Uncus	38
6.51	1.5	18.2	−19.3	R	F	Rectal Gyrus	11
5.73	50.8	−0.6	−28.2	R	T	Middle Temporal Gyrus	21
5.55	50.8	−33.7	−23.6	R	T	Fusiform Gyrus	20
5.31	60.6	−55	−17.6	R	O	Fusiform Gyrus	37
5.27	−58.5	−24.5	−15.5	L	T	Middle Temporal Gyrus	21
4.36	40.9	−75.2	−19.1	R	Cereb	Posterior Lobe, Declive	
3.66	1.5	−29.4	26	R	Limbic	Cingulate Gyrus	23
3.02	−38.5	2.4	29.4	L	F	Precentral Gyrus	6

## Discussion

The purpose of this study was to investigate the neural mechanisms underlying the human ability to understand emotional body language (EBL). To accomplish this goal, whole-figure photographs of 8 female and male actors portraying 40 typical emotional or mental states (e.g., “I am in love”, “I admire you so much!”, “I hate you” etc.) were taken. During the EEG recording sessions, each of 280 pictures was presented and preceded by a short verbal description of a feeling; this feeling was strongly incongruent with the content of the picture in half of the presentations. Behavioral and ERP data elicited by congruent and incongruent EBL displays were compared. To exclude the possibility that differences emerged due to discrepancies in purely sensory characteristics, all photographs were taken in the same conditions and were equiluminant, identical in size, and similar in many perceptual characteristics (e.g., each actor was present in the same number of congruent and incongruent trials).

Due to this careful balancing of perceptual factors, the electrophysiological signals showed no differences in the first 250 ms of visual processing (i.e., the P1 and N170 components) between the 2 classes of stimuli; this lack of difference is clearly illustrated in the ERP waveforms in figure 3 that were recorded at the occipitotemporal and lateral occipital sites. The lack of effects in the early P1 and N1 components demonstrates that the only difference between the two classes of photographs was their congruence with the preceding verbal definitions.

The earliest recognition of body language was indexed by the centroparietal P300 component, which was larger in response to congruent behavior in the time window between 280 and 440 ms. This congruence effect was more evident over the right visual area (i.e., PPO1 and PPO2), which most likely reflects the recognition activity (or priming effect) of cortical body- and face-devoted areas.

Previous studies of congruent/incongruent actions (e.g., [23], [24], [28]) have not reported posterior P3 and LP responses. This discrepancy is most likely due to methodological differences. In the present study, categorization based on action congruency was required of the participants, which generated P300-like responses to the congruent items; the tasks used in the aforementioned previous studies were implicit and involved secondary tasks that were not based on action categorization. Presumably, in these studies, action incongruence was automatically detected by the action-observation system, which generated anterior N400 responses to incongruent items, and no response-related P3 was generated. Indeed, Shibata and coworkers [27] observed large P300 responses to congruent actions when they asked their participants to evaluate the appropriateness of cooperative actions between two people.

Regarding the present investigation, the earliest increase in ERP amplitude in response to incongruent body language was observed at frontal, particularly inferior frontal, sites (F1, F2) in the time window of 380 to 460 ms and occurred in the form of a N400 deflection. The N400 component typically represents a supramodal index of conceptual processing and reflects difficulty in integrating incoming information with previously acquired information (in this case, verbal descriptions of emotional or mental states).

Previous ERP literature has revealed which neural circuits are involved in the recognition of purposeful versus purposeless behavior. It is thought that the activities of these circuits are reflected on the modulation of the anterior N400 response [18], [21]–[23], [27]. For example, Proverbio & Riva [22] provided evidence of that incongruent actions (e.g., a surgeon dissecting a book) elicit larger anterior negativities (i.e., N400) than do congruent actions (e.g., a woman doing the laundry), especially at inferior frontal sites (F1, F2). Indeed, the N400 response is not only sensitive to semantic and conceptual linguistic information but is also sensitive to violations of world-knowledge and communicative gestures [30]. Deaf native signers are especially sensitive to semantic violations and produce larger N400 responses than non-deaf controls [31]. Interestingly, Proverbio and coworkers [24] found that perceptions of incorrect basketball scenes elicited enlarged N400 responses at anterior sites in the 450–530 ms time window in skilled brains (i.e., professional basketball players). This deflection was totally absent in people who were unfamiliar with basketball. The modulation of the anterior N400 (which was larger at lateral anterior frontal sites; i.e., AF7 and AF8) was interpreted to reflect difficulty integrating incoming visual information with related sensorimotor knowledge. In this study [24], only professional basketball players detected violations in the system of basketball rules (i.e., violations of body postures, gestures, actions, or positions). A swLORETA inverse solution applied to the difference waves recorded in response to incorrect actions minus correct actions revealed that the strongest foci of activation were in the right temporal cortex, the inferior and superior temporal gyri (STG BA38), the right fusiform gyrus and the lingual gyrus (BA18). The lateral occipital area, also called the extrastriate body area (EBA) [32], is part of both the perception and action systems. Additionally, the superior temporal sulcus (STS) contains neurons that respond to the observation of biological actions such as grasping, looking or walking. In addition to visual areas, the perception of incorrect actions stimulated the right inferior parietal lobule (BA39/40), the precentral and premotor cortices (BA6), and the cerebellum of basketball players. The inferior parietal lobule has been shown to code transitive motor acts and meaningful behavioral routines (e.g., brushing teeth or flipping a coin). Indeed, lesions of the inferior parietal lobule are associated with impairments in the ability to recognize or perform skilled actions (such as lighting a cigarette or making coffee), and this deficit is called apraxia. In both groups, pictures of players in action strongly activated the right fusiform gyrus (BA37), a region that may include both the fusiform face area (FFA) [33] and the fusiform body area [34], which are regions that are selectively activated by human faces and bodies, respectively.

Moreover, in the present study, the analysis of the inverse swLORETA solution applied to the brain responses elicited by congruent and incongruent affective body language yielded a series of common activations that included the fusiform and the medial temporal gyri, which reflect the involvement of the activities of areas dedicated to the analyses of faces and bodies, such as the FFA, the fusiform body area (FBA) and the EBA. Additionally, we also found common activation of the parahippocampal gyrus, and this finding agrees with a similar finding of Proverbio and coworkers’ [24] aforementioned study on basketball players. Indeed, the parahippocampal gyrus might be involved in the visuospatial processing of places and analysis of spatial positions and orientations of body parts with respect to the space and environment [35], [36].

In the present study, the activation of the left superior frontal gyrus was associated with the processing of incongruent body language, while the same area was bilaterally activated during the perception of congruent body language. In congruent EBL conditions, activities in regions that are part of the fronto-parietal system have also been detected [37]; these regions include the left postcentral gyrus (BA3) and the right supramarginal gyrus (BA40) (the latter is also involved in coding incongruent body language). In contrast, the source in the left precentral gyrus (BA6) was found to be active only in response to incongruent EBL. This region is thought to play a crucial role in representing the goals of actions and the intentions of agents and has also been found to be active in previous studies of action recognition [23], [24], [28].

The swLORETA applied to the difference between congruent and incongruent EBL in the N400 time window revealed significant bilateral activity in the uncus, the anterior portion of the parahippocampal gyrus (BA28, BA38), and the right posterior cingulate cortex (BA 23); these regions belong to the limbic circuit involved, which is involved in emotional processing. These localizations agree with a large body of literature that indicates the primary involvements of the prefrontal and orbitofrontal cortices, the hippocampus [38] and the cingulate cortex [23] in emotional processing and the subjective evaluation of events and their significance [39]–[41]. In a recent study by Proverbio and coworkers [28] the processing of social cooperative and affective interactions were contrasted, which revealed a strong activation of the limbic system, especially the right posterior cingulate cortex, in response to purely affective interactions in the time window between 150 and 190 ms (corresponding to the N170 ERP response). Additionally, the involvement of the posterior cingulate cortex (BA23) in the recognition of appropriate (vs. inappropriate) actions has been reported by Proverbio and co-workers [23], especially in the brain of women, displaying a more emotional than rational reaction to action incongruence. Therefore, it seems that the cingulate cortex (along with other cortical regions including the inferior parietal area) is heavily involved in the mechanisms of empathy and promotes connections between the mirror system and the ability to infer the emotions and mental states of others [42], [43].

In our opinion, one of the most important results of the present study is that the strongest source of activity of the incongruent/congruent difference was located in the right rectal gyrus (BA11) of the ventromedial orbitofrontal cortex, which is located at the base of the frontal lobe and rests on the upper wall of the orbital cavity. This region is involved in the processing of social and emotional decisions and appears to be important for developing, evaluating and filtering emotional information. A region with these characteristics would be crucial for the recognition and processing of affective action content but not the goals of actions. Notably, our previous experiments investigating the comprehension of non-affective goal directed behavior did not implicate this region [22]–[24], which suggests that the specific role of this area is related to the processing of affective cues conveyed by body language.

The early anterior N400 was partly paralleled and followed by a centroparietal N400 that peaked between 400 and 600 ms in response to incongruent EBL and by a posterior LP over right visual areas that was larger in response to congruent EBL. The topographic distribution of the N400 was similar to that of typical central-parietal N400 responses that have been reported in verbal [44] and nonverbal language studies [18]. Consistent with our study, Gunter and Bach [18] observed a frontal N300 that was followed by a centro/parietal N400 response, and the latter response was larger following incongruent gestures. The centroparietal N400 is a supramodal multisensory component that is thought to reflect difficulty in integrating incoming inputs with previous information at a conceptual level that is independent of sensory modality. Classically, the N400 has been elicited by semantically anomalous incongruent words [45], but the N400 has also been elicited by incongruent/unexpected or infrequent/incomprehensible items presented as drawings [46], spoken or written language, pictures, and videos [47], [48]. An interesting ERP study found an anterior N3 that was followed by a centro/parietal N400 [25] (Van Elk et al., 2008); in this study, the subjects prepared meaningful or meaningless actions that were performed with objects and provided semantic categorization responses before executing the actions. Interestingly, the scalp distribution of the N400 effects for action-related body parts (the words *eye* and *mouth*) for meaningful actions was different than that of the effects of action-unrelated body parts. More specifically, a classical N400 effect with a posterior distribution was found for the comparison between action-unrelated and action-related body parts, whereas an anterior N400 effect was found for object-incongruent compared to object-congruent words.

It has been noted that the N400 tends to have a more anterior distribution when elicited by pictures or actions than when elicited by words [22], [49]–[51]. These anterior negativities in the range of the N400 are assumed to reflect image- or action-specific semantic processing that is functionally similar to the processing of amodal semantic information that is indexed by the linguistic centro parietal N400. According to Amoruso and coworkers [48] the activation of motor and premotor regions during action comprehension could partially explain the frontal distribution of N400 responses to incongruent body patterns or movements actions that have been observed in action processing studies.

Previous studies have linked the emerging of an anterior N400 to incongruent gestures as reflecting the activation of motor/premotor regions representing action intentions (see Proverbio & Riva [22] for a review). More specifically, previous source localization data indicated premotor, motor, inferior parietal cortices, and orbito-frontal cortex as possible neural generators of these effects [23]. In the present sudy, in which observers had to process the emotional state of the acting person, LORETA solution explaining N400 difference-wave (cong.-incong.) pointed out an intense activity in the so-called emotional brain (limbic system and orbito-frontal cortex), plus in the premotor cortex, involved in the processing of the action’s meaning, consistently with previous neuroimaging studies using neutral actions, or solely hand-actions.

As for behavioral performance, in this study, accuracy data showed how it was easier to exclude that a EBL display was paired to an incongruent verbal description (2% of errors), rather than establishing a correspondence with the congruent pair (12% of errors). Although speed of response was the same, uncertainty was higher for congruent than incongruent trials. A similar pattern of results was found by Lima and coworkers [52], in which action/gestures mismatches were recognized more accurately than action/gestures matches. However, findings from other studies are not consistent with this pattern, it depending on task requirements. For example, in Gu and coworkers’ study [53], whose task was to recognize a facial expression by choosing among one of 6 Ekman’s emotional category, participants made significantly better and faster decisions when the faces were accompanied by bodies with congruent expressions than when they were accompanied by bodies with incongruent expressions. Indeed, in their case the match decision was based on a choice between 6 possibilities (anger, fear, surprise, disgust, happiness, sadness), whereas in our experiment the number of possibilities was unknown and unpredictable. Ultimately, it is not rare to find a better performance for mismatch than match decisions. In a very interesting fMRI study [54], in which participants encoded the association between a person’s face and their home, and thereafter were asked to decide about the pair congruency, accuracy was found to be higher on mismatch than match trials. Importantly, the activity of CA_1_ region of hippocampus was significantly greater for correct mismatch (correct rejections) than match (hits) trials. Indeed, activation of CA_1_ was greater when participants encountered house-probes that violated their mnemonic predictions (correct mismatch) relative to probes that confirmed these predictions (correct match), thus providing the neural explanation of the increased behavioral performance for incongruent trials, as in our study.

In conclusion, the present results support previous findings regarding non-affective action processing [22]–[24], [28], [55]–[58] and report an activation of the frontoparietal system. Additionally, these results provide new evidence for the crucial role of the limbic and ventromedial orbitofrontal cortices in the recognition of emotional body language (EBL).

The ERP results indicate that face and body mimics undergo a prioritized processing (as early as 300 ms) that heavily involves the affective brain and that the output of this processing is rapidly compared with verbal information, which allows for regulation of communicative and social behavior that takes into account both linguistic and non-verbal cues. In this view, considering that we are conscious of our environment about half second past reality events (a person’s move, for example), the automatic processing of possible affective body signals at about 400 ms can be considered quick, and especially useful.
